# Motivational Interviewing Technique As a Means of Decreasing Vaccine Hesitancy in Children and Adolescents During the COVID-19 Pandemic

**DOI:** 10.1192/j.eurpsy.2023.1555

**Published:** 2023-07-19

**Authors:** P. A. Bidkhanian

**Affiliations:** Psychiatry, BronxCare Health System, Bronx, United States

## Abstract

**Introduction:**

Vaccine hesitancy is a known phenomenon predating the COVID-19 pandemic. Vaccine hesitancy is a significant factor effecting the control and spread of the COVID-19 Virus. Hesitancy of parents choosing not to vaccinate their children is studied here. Also studied is the effect of a brief motivational interviewing intervention on the parent’s decision to vaccine their child, or not. What was found was a myriad of beliefs and values in the parents, and varied reactions and outcomes in response to the motivational interviewing.

**Objectives:**

The aim of our study is to determine whether motivational interviewing techniques can be used as an effective tool to educate patients and their families about the benefits of vaccination against COVID-19 and increase vaccinations rates. In our urban community hospital-based child and adolescent psychiatry outpatient clinic, we found a high level of vaccine hesitancy among our patient population. As motivational interviewing is an evidence-based approach to addressing ambivalence and behavior change, we sought to engage parents with this approach.

**Methods:**

This is a quality improvement project where chart review of all pediatric patients currently enrolled in our clinic was performed to determine which patients are unvaccinated for COVID-19. Telephone contact was made to reach parents to obtain verbal consent and to deliver the intervention consisting of standardized motivational interview techniques followed by brief educational points about the vaccines. Follow up calls were made one week later to determine whether there was any change in readiness to consider vaccination on a scale from 1-10. We reached a total of 29 parents on initial outreach, and 11 parents during the follow up phase. Many were lost to follow up due to unavailability or refusal to participate further.

**Results:**

Parents (*N* = 11, 5- African American, 6- Hispanic) reported on their decision to have their child (mean age=12.2, 55% Female) receive the covid-19 vaccine. Preliminary findings show an increase in readiness on a scale from 1-10 to receive the vaccine following a one week interval post intervention (t(10)=2.096, p=.06), with the most common barriers that subjects endorsed being fear of side effects, skepticism regarding the speed at which the vaccines were developed, and wanting to allow their children to decide for themselves.

**Conclusions:**

We found that there was an overall improvement in vaccine hesitancy following our intervention, though it did not cross the threshold of statistical significance. We also identified common reasons given for hesitancy within our community. Motivational interviewing is a promising intervention to address vaccine hesitancy. Further study is warranted as expanding the reach of such interventions could lead to more robust data as well as broader vaccine acceptance.

**Disclosure of Interest:**

None Declared

